# Real-Time Control of an Articulatory-Based Speech Synthesizer for Brain Computer Interfaces

**DOI:** 10.1371/journal.pcbi.1005119

**Published:** 2016-11-23

**Authors:** Florent Bocquelet, Thomas Hueber, Laurent Girin, Christophe Savariaux, Blaise Yvert

**Affiliations:** 1 INSERM, BrainTech Laboratory U1205, Grenoble, France; 2 Univ. Grenoble Alpes, BrainTech Laboratory U1205, Grenoble, France; 3 CNRS, GIPSA-Lab, Saint-Martin-d'Hères, France; 4 Univ. Grenoble Alpes, GIPSA-Lab, Saint-Martin-d'Hères, France; 5 INRIA Grenoble Rhône-Alpes, Montbonnot, France; University of Buenos Aires, ARGENTINA

## Abstract

Restoring natural speech in paralyzed and aphasic people could be achieved using a Brain-Computer Interface (BCI) controlling a speech synthesizer in real-time. To reach this goal, a prerequisite is to develop a speech synthesizer producing intelligible speech in real-time with a reasonable number of control parameters. We present here an articulatory-based speech synthesizer that can be controlled in real-time for future BCI applications. This synthesizer converts movements of the main speech articulators (tongue, jaw, velum, and lips) into intelligible speech. The articulatory-to-acoustic mapping is performed using a deep neural network (DNN) trained on electromagnetic articulography (EMA) data recorded on a reference speaker synchronously with the produced speech signal. This DNN is then used in both offline and online modes to map the position of sensors glued on different speech articulators into acoustic parameters that are further converted into an audio signal using a vocoder. In offline mode, highly intelligible speech could be obtained as assessed by perceptual evaluation performed by 12 listeners. Then, to anticipate future BCI applications, we further assessed the real-time control of the synthesizer by both the reference speaker and new speakers, in a closed-loop paradigm using EMA data recorded in real time. A short calibration period was used to compensate for differences in sensor positions and articulatory differences between new speakers and the reference speaker. We found that real-time synthesis of vowels and consonants was possible with good intelligibility. In conclusion, these results open to future speech BCI applications using such articulatory-based speech synthesizer.

## Author Summary

Speech Brain-Computer-Interfaces may restore communication for people with severe paralysis by decoding cortical speech activity to control in real time a speech synthesizer. Recent data indicate that the speech motor cortex houses a detailed topographical representation of the different speech articulators of the vocal tract. Its activity could thus be used in a speech BCI paradigm to control an articulatory-based speech synthesizer. To date, there has been no report of an articulatory-based speech synthesizer producing fully intelligible speech, as well as no evidence that such device could be controlled in real time by a naive subject. Here, we present an articulatory-based speech synthesizer and show that it could be controlled in real time by several subjects articulating silently to produce intelligible speech.

## Introduction

In the past decades, brain-computer interfaces (BCIs) have been developed in order to restore capabilities of people with severe paralysis, such as tetraplegia or locked-in syndrome. The movements of different effectors, like a computer mouse or a robotic arm, were successfully controlled in several BCI studies, with increasing precision [1–9]. In the case of severe paralysis including aphasia (e.g., locked-in syndrome), ways of communicating can be provided by BCI approaches, mostly through a letter selection or typing process [1,10,11]. However, speech remains our most natural and efficient way of communication. BCI approaches could thus also be applied to control a parametric speech synthesizer in real-time in order to restore communication by decoding neural activity from speech processing brain areas [12,13]. Such perspective is indeed supported by an increasing number of studies reporting encouraging performances in decoding speech utterances, including phones, words or even full sentences, from brain activity [14–21].

Several brain areas are involved in speech processing, forming a wide cortical network, classically modeled by a ventral and a dorsal stream [22,23]. Because this speech network is widely distributed, a choice needs to be made on the cortical areas where to extract and decode signals for a speech BCI. One possibility is to probe signals from auditory areas, which encode the spectro-temporal representation of speech. However, auditory areas are widely involved in the sensory perception and integration of all sounds a person is exposed to, including speech and non-speech environmental sounds. For this reason, probing neural activity in areas more specifically dedicated to speech production might be more relevant for conversational speech production with BCI. It should be noted that although aphasia caused by strokes very often affect the articulatory speech motor cortex or other cortical areas necessary for speech production, this is not the case for other types of aphasia, such as in locked-in patients or patients with amyotrophic lateral sclerosis, for whom cortical speech activity can be intact or largely conserved and thus exploitable in a BCI perspective. In such case, and if successful, continuous speech restoration would be more beneficial than an indirect communication scheme such as a letter selection process.

Previous anatomical and functional data indicate that the speech sensorimotor cortex exhibits a somatotopic organization mapping the different articulators involved in speech production [24–28], the detailed dynamics of which can be highlighted using high-density neural recording [21]. Interestingly, it was further showed recently that during speech production, the activity of the speech sensorimotor cortex is rather tuned to the articulatory properties of the produced sounds than to their acoustic properties [29]. While neural data could be decoded directly into acoustic parameters, these data thus support our hypothesis that a relevant strategy could be to consider a more “indirect” approach accounting for the articulatory activity of the vocal tract under control of the speech sensorimotor cortex to produce sounds. In such approach, cortical signals will be probed and decoded to control in real time a parametric articulatory-based speech synthesizer having enough degrees of freedom to ensure continuous intelligible speech production. Interestingly, these articulatory features are generally considered lower dimensional and varying more slowly in time than acoustic features, thus possibly easier to predict from cortical signals.

Articulatory-based speech synthesizers are able to generate an intelligible speech audio signal from the position of the main speech articulators: tongue, lips, velum, jaw, and larynx [30–36]. Articulatory-based synthesizers are mainly divided into two categories: physical or geometrical approaches (such as in [36–39]), which aim to model the geometry of the vocal tract and its physical properties, and machine-learning approaches (such as [34,35]), which use large databases to automatically learn the mathematical relationship between articulatory and acoustic data. Here we made the choice to build a synthesizer using a machine-learning approach. Indeed, to synthesize speech, geometrical and physical models must first solve the difficult inverse problem of recovering articulatory parameters from the acoustic of each calibration sequence. By contrast, with machine-learning approaches, a large articulatory-acoustic database must be recorded, thus avoiding this issue for all the sentences of this database. For BCI applications, one will require a parallel dataset of brain signals and control parameters for the synthesizer: here we can use known data from the articulatory-acoustic database. Moreover, geometrical and physical models need high computation power while, once trained, a machine-learning model is very fast to apply for real-time synthesis. Finally, we showed in a previous study that such machine-learning approach is robust to noisy input parameters [35], which is a non-negligible asset for BCI applications, when the decoding of neural data results in non-perfect signals.

Interestingly, articulatory-based synthesizers can be controlled with about 10 continuous parameters [35,40], which is of the order of the number of degrees of freedom controlled simultaneously in recent complex motor BCI paradigms in monkeys [41] and human participants [9,42].

However, it remains unknown whether a given articulatory-based speech synthesizer built from articulatory-acoustic data obtained in one particular reference speaker can be controlled in real time by any other speaker to produce intelligible speech. In this context, we present here an articulatory-based speech synthesizer producing intelligible speech that can be controlled in real time for future BCI applications. This synthesizer is based on a machine-learning approach in which the articulatory data recorded by electro-magnetic articulography (EMA) is converted into acoustic speech signals using deep neural networks (DNNs). We show that intelligible speech could be obtained in a closed-loop paradigm by different subjects controlling this synthesizer in real time from EMA recordings while articulating silently, i.e. without vocalizing. Such a silent speech condition is as close as possible to a speech BCI paradigm where the synthetic voice replaces the actual subject voice. These results thus pave the way toward a future use of such articulatory-based speech synthesizer controlled by neural activity in a speech BCI paradigm.

## Materials and Methods

### Ethics Statement

All subjects gave their informed consent to participate in the study, which was approved by the local ethical committee of Grenoble for non-interventional research (CERNI) under approval No. 2016-01-05-82.

### Subjects

Four French native speakers (1 female, 3 males) participated in the study. One male subject was the reference speaker from whom data the synthesizer was built, and all four subjects then controlled in real time the synthesizer.

### Construction of an articulatory-based speech synthesizer

**Fig 1:**
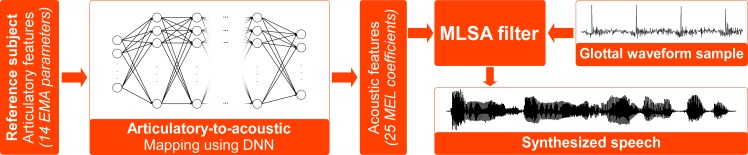
Articulatory-based speech synthesizer. Using a DNN, articulatory features of the reference speaker are mapped to acoustic features, which are then converted into an audible signal using the MLSA filter and an excitation signal.

In a first step, we designed an intelligible articulatory-based speech synthesizer converting the trajectories of the main speech articulators (tongue, lips, jaw, and velum) into speech (see Fig 1). For this purpose, we first built a large articulatory-acoustic database, in which articulatory data from a native French male speaker was recorded synchronously with the produced audio speech signal. Then computational models based on DNNs were trained on these data to transform articulatory signals into acoustic speech signals (i.e. articulatory-to-acoustic mapping). When considering articulatory synthesis using physical or geometrical models, the articulatory data obtained by EMA can be mapped to the geometrical parameters of the model [39]. Here we consider a machine-learning approach in which the articulatory data obtained by EMA is directly mapped to the acoustic parameters of a vocoder.

#### Articulatory data acquisition and parameterization

**Fig 2:**
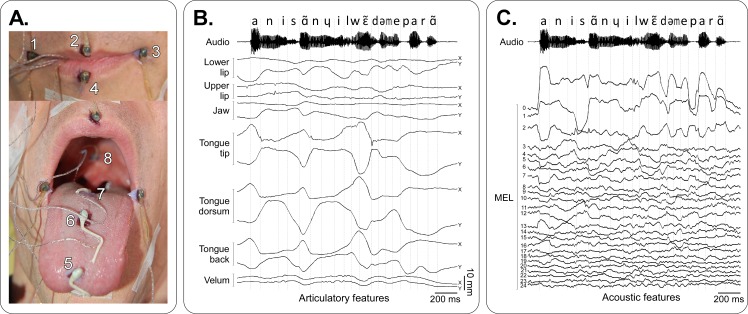
Articulatory and acoustic data. **A**–Positioning of the sensors on the lip corners (1 & 3), upper lip (2), lower lip (4), tongue tip (5), tongue dorsum (6), tongue back (7) and velum (8). The jaw sensor was glued at the base of the incisive (not visible in this image). B–Articulatory signals and corresponding audio signal for the sentence “Annie s’ennuie loin de mes parents” (“Annie gets bored away from my parents”). For each sensor, the horizontal caudo-rostral X and below the vertical ventro-dorsal Y coordinates projected in the midsagittal plane are plotted. Dashed lines show the phone segmentation obtained by forced-alignment. C–Acoustic features (25 mel-cepstrum coefficients—MEL) and corresponding segmented audio signal for the same sentence as in B.

The articulatory data was recorded using the electromagnetic articulography (EMA) NDI Wave system (NDI, Ontario, Canada), which allows three-dimensional tracking of the position of small coils with a precision of less than a millimeter. Nine such 3D coils were glued on the tongue tip, dorsum, and back, as well as on the upper lip, the lower lip, the left and right lip corners, the jaw and the soft palate (Fig 2A). This configuration was chosen for being similar to the ones used in the main publicly available databases, such as MOCHA (http://www.cstr.ed.ac.uk/research/projects/artic/mocha.html) and mngu0 [43], and in other studies in articulatory-based synthesis [34] or articulatory-to-acoustic inversion [44]. This configuration allows to capture well the movements of the main articulators while avoiding to perturb the speaker too much: 3 coils on the tongue give information on back, dorsum and apex while 4 coils on lips give information on protrusion and rounding, and we considered that one sensor was enough for the jaw since it is a rigid articulator, and one for the soft palate since it has mostly one degree of freedom. An additional 6D reference coil (which position and orientation can be measured) was used to account for head movements and was glued behind the right ear of the subject. To avoid coil detachment due to salivation, two precautions were taken to glue the sensors. First, the tongue and soft palate sensors were glued onto small pieces of silk in order to increase contact surface, and second, the tongue, soft palate and jaw surfaces were carefully dried using cottons soaked with 55% green Chartreuse liquor. The recorded sequences of articulatory coordinates were down-sampled from 400 Hz to 100 Hz.

Finally, in order to assess the effect of the number of articulatory parameters on the intelligibility of synthesized signals, four different parameterizations of the articulatory data were used: using the raw 3D data of the 9 sensors (27 parameters), projecting the data in the midsagittal plane and removing the lip-corner sensors (14 parameters, see Fig 2B), and reducing the original data to either 10 or 7 parameters using principal component analysis (PCA).

#### Acoustic data acquisition, parameterization and synthesis

The speech signal was recorded at 22,050 Hz synchronously with the articulatory data. Its spectral content was parameterized by 25 mel-cepstrum (MEL) coefficients (Fig 2C) computed every 10 ms (hence a 100 Hz sampling matching the articulatory data acquisition frequency) from a 23-ms (512 samples) sliding window using the Speech Processing ToolKit (SPTK, http://sp-tk.sourceforge.net/) *mcep* tools [45]. These 25 coefficients efficiently represent the spectral envelope of speech and can be converted back into audible sounds by building a so-called Mel Log Spectrum Approximation (MLSA) filter [46]. This approach is based on the source-filter model of speech production, which models the speech signal as a convolution of a sound source (e.g., the glottal activity) with a linear acoustic filter representing the vocal tract,. In the present MLSA model, a set of M mel-cepstrum coefficients *c*_*α*_(*m*) represent the vocal tract filter *H*(*z*) for each audio signal window, as follows:

$$H(z)=exp\;\sum_{m=0}^{M}c_{\alpha }(m).{\tilde{z}}^{-m},$$

$$with\;{\tilde{z}}^{-1}=\frac{z^{-1}-\alpha }{1-\alpha z^{-1}}.$$

The coefficient α is chosen so that the mel-scale becomes a good approximation of the human sensitivity to the loudness of speech (here α = 0.455). The mel-cepstral coefficients *c*_*α*_(*m*) are approximated using the Newton-Raphson method for numerically solving equations, and linearly combined to obtain the MLSA filter coefficients. This filter is then excited with a source signal representing the glottal activity (i.e. vibration of the vocal folds). Such excitation signal is generally designed by extracting the pitch from the original audio signal, and then generating white noise for non-voiced segments, and a train of pulses for voiced-segments, which period varies according to the pitch. However, since there is no such glottal activity in silent speech, we as well designed an artificial template-based excitation signal using the glottal activity from a single vowel /a/. While such glottal activity could be recorded using an electroglottograph as in [47], here we estimated it using inverse filtering [48] (using the SPTK *mlsadf* tool). Using such an excitation signal results in an unnatural (‘‘robotic”) speech sound in which all the phonemes are voiced, and have the same pitch. Transcription of the audio signals was first done manually in naturally written text, then translated into phone sequences using *LLiaPhon phonetizer* (https://gna.org/projects/lliaphon) [49], and finally manually corrected. Phone sequences were then automatically aligned on audio files using a standard speech recognition system (based on a set of tied-state context-dependent phonetic hidden Markov models (HMM) trained using the HTK toolkit http://htk.eng.cam.ac.uk/)) and a forced-alignment procedure.

#### Reference articulatory-acoustic database (the BY2014 corpus)

**Fig 3:**
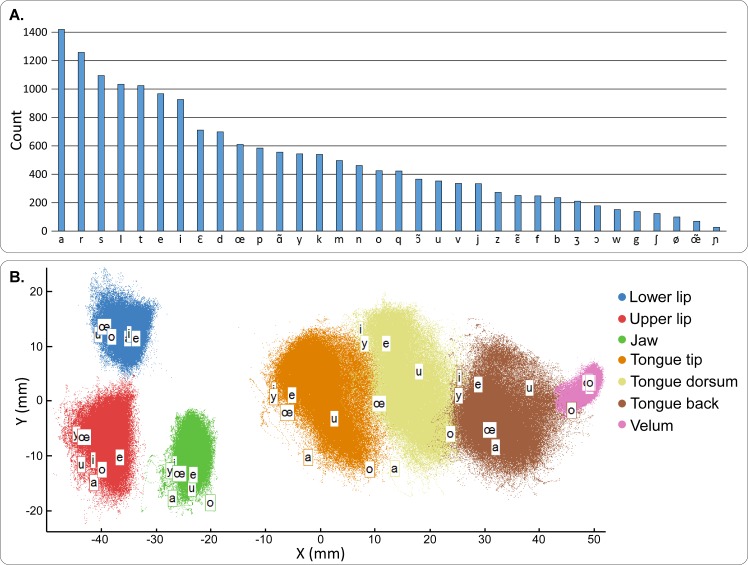
Articulatory-acoustic database description. A–Occurrence histogram of all phones of the articulatory-acoustic database. Each bar shows the number of occurrence of a specific phone in the whole corpus. B–Spatial distribution of all articulatory data points of the database (silences excluded) in the midsagittal plane. The positions of the 7 different sensors are plotted with different colors. The labeled positions correspond to the mean position for the 7 main French vowels.

For this specific study, we recorded a large articulatory-acoustic database, named BY2014, containing more than 45 minutes of speech after removing the periods of silence. This database was composed of 712 items of variable length, ranging from isolated vowels, vowel-consonant-vowel sequences (VCVs), phonetically balanced sentences, and other sentences extracted from French newspaper “Le Monde”. This resulted in 18,828 phones in total. The distribution of the 34 different phonetic classes used to describe French language in this corpus is shown in Fig 3A. Phone frequency ranged from 1,420 occurrences for the phone /a/ to 27 occurrences for /ɲ/. The distribution of the articulatory data points in the midsagittal plane is represented in Fig 3B. The velum was the articulator with the smallest movement amplitude (less than 1cm), followed by the jaw and the upper lip (about 1cm), the lower lip (about 2cm), and finally the tongue had the highest amplitude of movement (about 3cm for each sensor). In the following, this dataset is referred to as the *reference data*, and the subject from whom this data was recorded is referred to as the *reference speaker*. This dataset is available for download at https://zenodo.org/record/154083 (doi: 10.5281/zenodo.154083).

#### Articulatory-to-acoustic mapping using Deep Neural Networks (DNN)

The articulatory-to-acoustic mapping was performed using a deep neural network (DNN) trained on the reference data (see Fig 1). A DNN is an artificial neural network with more than one or two hidden layers, which can be used to address complex (and highly non-linear) classification and regression problems. More details can be found in [50]. The choice for using a DNN-based mapping was motivated by previous results [35,51] showing that such mapping was more robust to noisy input than a state-of-the-art mapping based on Gaussian Mixture Model (GMM) such as the one proposed by Toda et al. [34], and thus likely a better candidate for future BCI applications where input articulatory control signals will be inferred from noisy cortical signals.

Here, we considered a fully-connected feed-forward DNN with 3 hidden layers containing 200 leaky rectified linear units (LReLU), which are known to improve convergence of the training procedure and to lead to better solutions than conventional sigmoid units [52]. Each unit of a layer was connected to all units of the next layer, and there was no connection between units belonging to the same layer. Both input (articulatory) and output (acoustic) data were z-scored (subtraction of the mean and then division by the standard deviation) before being fed to the network, and data frames corresponding to silence periods were removed. To take into account the dynamic properties of speech, we concatenated each articulatory frame with its 4 preceding frames (50-ms time window context compatible with a real-time implementation). The DNN thus mapped the articulatory input features to 25 output mel-cepstrum coefficients, which were then converted into an audible speech signal using the MLSA filter and the excitation signal.

DNN training is usually a complex task since large initial weights typically lead to poor local minima, while small initial weights lead to small gradients making the training infeasible with many hidden layers [53]. Here, we trained our network using the classical back-propagation algorithm [54]. However, we chose to add the different layers successively. During the first step, the network was only composed of the input layer, the first hidden layer, and the output layer. This initial network was randomly initialized then fine-tuned using back-propagation. Then a new hidden layer was added and the output layer was replaced with a new one so that the new network was now composed by the input layer, the previously trained hidden layer, the new hidden layer, and the new output layer. The weights from the input layer to the first hidden layer were those obtained at the previous step and the other weights were randomly initialized. Back-propagation was then applied to this new network for fine-tuning. This process was repeated until all the hidden layers were added. At each step, the weights of a given layer were randomly initialized using a Gaussian distribution with a zero mean and a 1/N standard deviation, where N was the number of units of the previous layer. The error criterion was the mean squared error (MSE) between predicted and expected values:

$$MSE=\frac{\sum_{i=1}^{D}{\left(o_{i}-m_{i}\right)}^{2}}{D},$$

where *o*_*i*_ is the i-th output of the network, D = 25 is the number of MEL outputs, and *m*_i_ is the i-th z-scored MEL coefficient computed on the original audio data. The minimization of this error was done with the Polack-Ribière conjugate gradient method using 3 quadratic/cubic line searches [55], on successive batches: at each epoch, the training data samples were randomly shuffled and then divided into 100 blocks. Dividing into batches allowed more efficient computation than when using single samples [52]. The training was made by randomly selecting 90% of the articulatory-acoustic database items, while half of the remaining 10% were used for early stopping of the training (the training was stopped when the error on this validation set did not improve over 20 epochs) and the other half for testing. The whole training was done using custom-made optimization tools written in C++, the conjugate gradient code being adapted from the Matlab implementation of the DRToolBox (http://lvdmaaten.github.io/drtoolbox/). Four different DNNs were trained, one for each of the four different parametrizations of the articulatory data (with 27, 14, 10 and 7 articulatory parameters).

### Real-time control of the articulatory-based speech synthesizer

**Fig 4:**
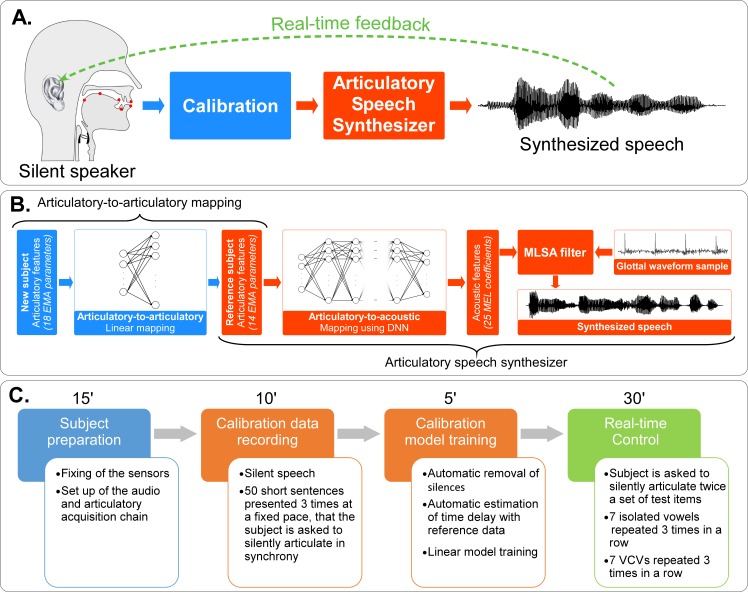
Real-time closed loop synthesis. A) Real-time closed-loop experiment. Articulatory data from a silent speaker are recorded and converted into articulatory input parameters for the articulatory-based speech synthesizer. The speaker receives the auditory feedback of the produced speech through earphones. B) Processing chain for real-time closed-loop articulatory synthesis, where the articulatory-to-articulatory (left part) and articulatory-to-acoustic mappings (right part) are cascaded. Items that depend on the reference speaker are in orange, while those that depend on the new speaker are in blue. The articulatory features of the new speaker are linearly mapped to articulatory features of the reference speaker, which are then mapped to acoustic features using a DNN, which in turn are eventually converted into an audible signal using the MLSA filter and the template-based excitation signal. C) Experimental protocol. First, sensors are glued on the speaker’s articulators, then articulatory data for the calibration is recorded in order to compute the articulatory-to-articulatory mapping, and finally the speaker articulates a set of test items during the closed-loop real-time control of the synthesizer.

In a second step, four speakers controlled the synthesizer in real time. As built, the synthesizer could only be used on the reference data and could not be directly controlled by another speaker or even by the same speaker in a different session. Indeed, from one session to another, sensors might not be placed at the exact same positions with the exact same orientation, or the number of sensors could change, or the speaker could be a new subject with a different vocal tract geometry and different ways of articulating the same sounds. In order to take into account these differences, it was necessary to calibrate a mapping from the articulatory space of each new speaker (or the same reference speaker in a new session) to the articulatory space of the reference speaker, that is, an articulatory-to-articulatory mapping (Fig 4A and 4B, left blue part). To achieve this calibration, we acquired articulatory data from the new speaker that corresponded to known reference articulatory data. This calibration model was then applied in real time to incoming articulatory trajectories of each silent speaker to produce continuous input to the speech synthesizer. Since the subjects were in silent speech and thus no glottal activity was available, we chose to perform the synthesis using the fixed-pitch template-based excitation, and in order to reduce the number of control parameters, we chose the synthesis model using 14 articulatory parameters since results showed that it was able to produce fully intelligible speech (see first part of the Results section). Fig 4C summarizes the whole experimental protocol, which is detailed in the following sections.

#### Subjects and experimental design

Four subjects (1 female, 3 males) controlled the synthesizer in real time. The reference speaker was one of them (Speaker 1), i.e. he was also used as a test subject, but with data from a different session than the reference data session. The articulatory data was recorded using the NDI Wave system in similar conditions as for acquiring the reference data (recording at 400Hz and down-sampling to 100Hz), except that only 6 sensors were used to record the articulatory movements of the lower and upper lips, the tongue tip, dorsum and back, and the jaw. This was due to the fact that the NDI Wave system was limited to 6 sensors when retrieving the articulatory data in real-time (while it was possible to use more sensors in offline mode). The soft palate sensor was one of the discarded sensor because most subjects (3 out of 4) were very uncomfortable with keeping the soft palate sensor for a long duration. The corner lip sensors were also discarded because they were the least informative for the synthesis. While the coils were positioned at the same anatomical locations, no particular attention was given to place them at very precise locations.

#### Articulatory-to-articulatory mapping

In order to estimate the articulatory-to-articulatory mapping, it was necessary to obtain articulatory data from the new speakers in synchrony with articulatory data from the reference speaker when articulating the same sounds. The new speakers were thus asked to silently repeat a subset of 50 short sentences (about 4 words each), extracted from the reference articulatory-acoustic database, in synchrony with the corresponding audio presented through earphones. Each sentence was first displayed on the screen during one second, then after a visual countdown, it was played three times at a fixed pace, so that the speaker could adapt to the reference speaker rate and way of speaking. Only the last repetition was considered in order to obtain the best temporal synchronization. Subjects were asked to repeat the sentences silently, and not loudly, because of significant differences that may exist between silent and vocalized speech, and because subsequent real-time closed-loop control of the synthesizer would then be achieved while subjects were silently speaking. For each silent speaker, the articulatory-to-articulatory mapping was performed using a linear model mapping the articulatory data of the speaker to those of the reference speaker. For this purpose, the frames corresponding to silence periods were discarded. In order to counterbalance speaker and system latencies, a global delay between new and reference articulatory data was estimated for each speaker by trying different delays. A different linear model between the new speaker’s articulatory data and the reference data was computed for each candidate delay. Each linear model was then applied to the new speaker’s articulatory data, and the mean-squared error (MSE) between predicted and actual reference articulatory data was computed. The delay which led to the smallest MSE was considered to be due to system latency and was corrected before the training of the final model by simply shifting and cutting the original data. Out of the 50 calibration sentences, 40 were used for the training of the articulatory-to-articulatory mapping while the remaining 10 sentences were kept for testing (randomly chosen, but identical across all speakers). By contrast with the articulatory-to-acoustic mapping, the articulatory-to-articulatory mapping was done frame-by-frame, without concatenating any past frame.

#### Real-time control

As shown in Fig 4B, the real-time control of the articulatory-based speech synthesizer was achieved by cascading the linear model used for articulatory-to-articulatory mapping and the DNN used for articulatory-to-acoustic mapping (using 14 articulatory parameters). The input articulatory data capture and processing (especially the re-referencing with regards to the reference sensor), the linear and DNN mappings and the MLSA filter were all implemented within the Max/MSP environment (Cycling’74, Walnut CA, USA, https://cycling74.com/products/max/) dedicated to real-time audio processing. Special attention was given to audio settings in order to minimize the audio chain latency and obtain a delay inferior to 30ms. Since the subjects were in silent speech condition, and thus no glottal activity was present, we used the template-based excitation signal for the MLSA filter. During this closed-loop situation, each speaker was asked to silently articulate a set of test items while given the synthesized auditory feedback through amagnetic Nicolet TIP-300 insert earphones (Nicolet Biomedical, Madison, USA) ensuring no interference with the magnetic field of the NDI Wave system. The auditory feedback was recorded for further intelligibility evaluation. Subjects were allowed to adapt to the closed-loop situation for at least 20 minutes. Then, they were asked to pronounce a set of test items, which were not part of the datasets used to train the articulatory-to-acoustic and the articulatory-to-articulatory mappings. The test set consisted of the 7 isolated vowels /a/, /e/, /i/, /o/, /u/, /œ/, and /y/, and 21 vowel-consonant-vowel (VCV) pseudo-words made by the 7 consonants /b/, /d/, /g/, /l/, /v/, /z/ and /ʒ/, in /a/, /i/ and /u/ context (e.g., ‘aba’ or ‘ili’). We chose not to include nasal vowels (e.g., /$\tilde{ɑ}$/, which corresponds to a nasalized /a/) since no sensor was placed on the soft palate. Likewise, we did not include nasal consonants (e.g., /m/ or /n/) and most unvoiced consonants (e.g., /p/ which roughly corresponds to an unvoiced /b/). Each item of the test set was repeated three times in a row. The whole set of items was repeated three times by each speaker, each repetition being separated by about 10 minutes of free control of the synthesizer.

### Evaluation protocol

The quality of open and closed-loop speech synthesis was assessed in two ways. We first carried out a qualitative evaluation, in which the acoustic signals obtained by offline synthesis from the reference database corpus (reference offline synthesis) or during closed-loop experiments (closed-loop synthesis) were compared with the original signals processed through analysis-synthesis. Analysis-synthesis was performed by converting the audio signals into mel-cepstrum (MEL) coefficients, which were then directly converted back into audio signals using the MLSA filter and template-based excitation. Such signal is referred here to as the ‘anasynth signal’. This conversion is not lossless, though it represents what would be the best achievable quality for the synthetic speech signal in the present context.

Then we carried out a quantitative evaluation of our system through an intelligibility test. Twelve subjects participated to this test. All participants were French native speakers with no hearing impairment.

For the offline reference synthesis, the evaluated stimuli consisted of the 10 vowels /a/, /i/, /u/, /o/, /œ/, /e/, /y/, /ã/, /$\tilde{ɛ}$/, and /$\tilde{ɔ}$/, and the 48 VCVs made of /p/, /t/, /k/, /f/, /s/, /ʃ/, /b/, /d/, /g/, /v/, /z/, /ʒ/, /m/, /n/, /r/, and /l/, in /a/, /i/ and /u/ contexts (i.e., ‘apa’, ‘iti’, ‘uku’, and so on). Each stimuli was synthesized and thus evaluated in 5 different conditions: 4 times using a pulse train excitation generated using the original pitch for each different number of articulatory parameters (27, 14, 10 and 7), and one time using the artificial template-based excitation signal (corresponding to a constantly voiced sound) with all 27 articulatory parameters. In the following, these 5 conditions are respectively denoted as *Pitch_27*, *Pitch_14*, *Pitch_10*, *Pitch_7* and *FixedPitch_27*. This allowed us to evaluate both the influence of the number of articulatory parameters on the intelligibility, and the effect of using or not using glottal activity information (here, the pitch). Indeed, while in the real-time closed-loop experiment presented here no glottal activity is recorded, this glottal activity could be obtained by decoding the neural activity in future BCI application. An additional evaluation was performed for the two conditions *Pitch_27* and *Pitch_14*, which consisted in directly transcribing 30 sentences (see S1 Appendix for the list of these sentences). For each listener, half of the sentences were randomly picked from the first condition and the other half from the other, ensuring that each listener never evaluated the same sentence twice, and that all sentences were evaluated in both conditions.

For the real-time closed-loop synthesis, the evaluated stimuli consisted of the 7 vowels /a/, /e/, /i/, /o/, /u/, /œ/, and /y/, and the 21 VCVs made of /b/, /d/, /g/, /l/, /v/, /z/ and /ʒ/, in /a/, /i/ and /u/ contexts. Each listener evaluated 3 repetitions (randomly picked for each listener) of each of these 28 items for each of the 4 new speakers. Remind that for the real-time closed-loop synthesis, the stimuli were generated using only the fixed-pitch template-based excitation.

In total, each listener had thus to identify 626 sounds (10 vowels + 48 VCVs for each of the 5 different offline synthesis conditions, 7 vowels + 21 VCVs, three times for each of the 4 speakers) and transcribe 30 sentences. The sounds were all normalized using automatic gain control, and played in random order at the same sound level through Beyerdynamic DT-770 Pro 80 Ohms headphones, while the listener was seated in a quiet environment. No performance feedback was provided during the test.

For the VCVs and vowels evaluation, participants were instructed to select in a list what they thought was the corresponding vowel in the case of an isolated vowel, or the middle consonant in the case of a VCV sequence. Graphical user interface buttons were randomly shuffled for each subject in order to avoid systematic default choice (e.g., always choosing the left button when unable to identify a sound). The subjects were told that some of the sounds were difficult to identify, and thus to choose the closest sound among the offered possibilities. The recognition accuracy was defined as *Acc = R/N* with *R* the number of correct answers for the *N* presented sounds of the test. Since each item had exactly the same number of repetitions, the chance level was estimated by *Acc*_*Chance*_ *= 1/C*, with *C* the number of different item categories. For the offline reference synthesis, the chance level was thus *1/10 = 10%* for vowels, and *1/16≈6%* for VCVs, while for the real-time closed-loop synthesis, the chance level was *1/7≈14%* in both cases.

For the sentences, the subjects were asked to transcribe directly the sentences they were listening to. Results were evaluated using the word accuracy *WAcc = (N—S—D—I)/N* (with *N* the total number of words, *S* the number of word substitutions, *D* the number of deletions and *I* the number of insertions), which is a commonly used metric in the field of automatic speech recognition.

### Statistical analysis

#### Analysis of the offline reference synthesis

Several listeners had to identify the same synthesized items, resulting in a binary answer (wrong or right), for each item and each listener. Statistical analysis of these results was thus performed using mixed logistic regression. For the VCVs and vowels, the following model was used: *Result ~ (Segment + Condition)^2 + (1 | Listener)*, where *Result* is the binary answer (equals 0 if the item was wrongly identified, otherwise 1), *Segment* has two levels corresponding to the type of item (vowel or VCV), *Condition* has five levels corresponding to the five different conditions (*Pitch_27*, *Pitch_14*, *Pitch_10*, *Pitch_7* and *FixedPitch_27*), and *Listener* has 12 levels corresponding to each listener that participated in the listening test. Multiple comparisons were made using contrasts according to [56]. For the sentences, a paired Student test was performed to compare the results. All the tests were made using the R software, and packages *lme4*, *multcomp* and *lsmeans*.

#### Analysis of the articulatory-to-articulatory mapping

For the articulatory-to-articulatory mapping, mean distance between predicted articulatory trajectories and reference articulatory trajectories was computed for each item of the test corpus and each speaker. A two-factor ANOVA with repeated measures was performed using the following model: Distance ~ Sensor*RefSpeaker + Error (Item / (Sensor*RefSpeaker)), where *Distance* is the mean distance between predicted and reference trajectories, *RefSpeaker* has two levels indicating if it was the reference speaker (Speaker 1) or another speaker (Speaker 2, 3 or 4), *Item* corresponds to the identifier of the tested item, and *Sensor* has 7 levels corresponding to the different EMA sensor positions to be predicted (upper lip, lower lip, jaw, tongue tip, tongue dorsum, tongue back and velum). Multiple comparisons were made using contrasts according to [56]. All the tests were made using the R software, and packages *lme4*, and *multcomp*.

#### Analysis of the real-time closed-loop synthesis

As for offline reference synthesis, statistical analysis of the real-time closed-loop synthesis results was performed using mixed logistic regression. The following model was used: *Result ~ (Segment + RefSpeaker) ^ 2 + (1 | Listener)*, where *Result* is the binary answer (equals 0 if the item was wrongly identified, otherwise 1), *Segment* has two levels corresponding to the type of item (vowel or VCV), *RefSpeaker* has two levels indicating if it was the reference speaker (Speaker 1) or another speaker (Speaker 2, 3 or 4), and *Listener* has 12 levels corresponding to each listener that participated in the listening test. Multiple comparisons were made using contrasts according to [56]. All the tests were made using the R software, and packages *lme4*, *multcomp* and *lsmeans*.

## Results

### Intelligibility of the articulatory-based speech synthesizer (offline reference synthesis)

**Fig 5:**
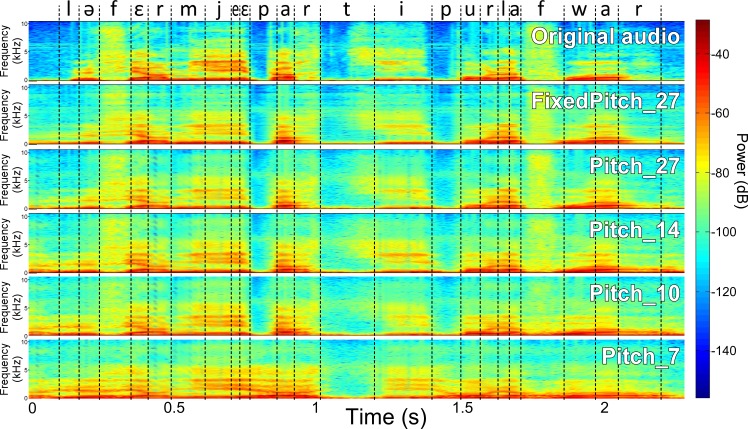
Offline reference synthesis example. Comparison of the spectrograms of the original audio, and the corresponding audio signal produced by the 5 different offline articulatory synthesis for the sentence “Le fermier est parti pour la foire” (“The farmer went to the fair”). Dashed lines show the phonetic segmentation obtained by forced-alignment.

First, we evaluated the proposed DNN-based articulatory synthesizer described in the Methods section. Fig 5 shows the spectrogram of the original sound for an example sentence (the sentence was *“Le fermier est parti pour la foire”*, meaning *“The farmer went to the fair”*), together with the 5 different synthesis. Note that there is speech present in the synthesized sample before the actual beginning of the reference sentence, since no assumption can be made on the presence of the air flow when considering only articulatory data. The corresponding synthesized sounds are provided in S1–S6 Audio Files, further illustrating the good intelligibility of the synthesized sounds when using at least 10 articulatory parameters. Note however that, in the following, the quality of the articulatory-to-acoustic mapping was evaluated subjectively by naive listeners mainly on isolated vowels and VCVs in order to avoid the influence of the linguistic context that tends to over-estimate evaluation results.

**Fig 6:**
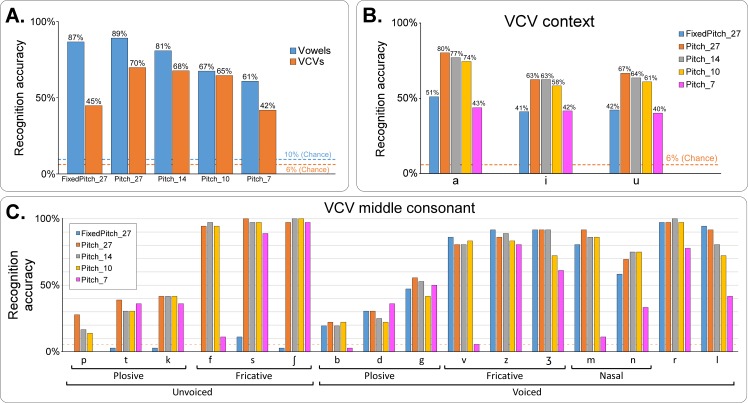
Subjective evaluation of the intelligibility of the speech synthesizer (offline reference synthesis). **A**–Recognition accuracy for vowels and consonants for each of the 5 synthesis conditions. The dashed lines show the chance level for vowels (blue) and VCVs (orange). B–Recognition accuracy of the VCVs regarding the vocalic context, for the 5 synthesis conditions. The dashed line shows the chance level. C–Recognition accuracy of the consonant of the VCVs, for the 5 synthesis conditions. Dashed line shows the chance level. See text for statistical comparison results.

Fig 6 summarizes the result of the subjective listening test. The recognition accuracy was better for vowels than for consonants for *FixedPitch_27*, *Pitch_27* and *Pitch_7* (*P* < 0.01), while this difference was only a trend for *Pitch_14* (*P* = 0.0983) and *Pitch_10* (*P* > 0.99).

For vowels, the recognition accuracy was far above chance (chance level = 10%) for all conditions (*P* < 0.01, Fig 6A) and decreasing when decreasing the number of articulatory parameters, ranging from 89% for *Pitch_27* to 61% for *Pitch_7*. Taking *Pitch_27* as reference, this decrease was not found significant for *Pitch_14* (*P* = 0.7116), and significant for *Pitch_10* and *Pitch_7* (*P* < 0.01 in both cases). No statistically significant difference was observed when not using the glottal activity versus when using the glottal activity (*FixedPitch_27* = 87%, *Pitch_27* = 89%, *P* > 0.99).

For the consonants, the recognition accuracy was also far above chance (chance level = 6.25%) for all conditions (*P* < 0.01, Fig 6A). A decrease in recognition accuracy was also observed when decreasing the number of articulatory parameters, ranging from 70% for *Pitch_27* to 42% for *Pitch_7*. However, taking *Pitch_27* as reference, this decrease was not significant for *Pitch 14* (*P* > 0.99) and *Pitch 10* (*P* = 0.6328), and only significant for *Pitch_7* (*P* < 0.01). A significant difference was observed when not using the glottal activity (*FixedPitch_27* vs *Pitch_27*, *P* < 0.01). The differences in recognition accuracy for each condition were studied regarding the vowel of the VCV (Fig 6B) and the consonant (Fig 6C). Overall the intelligibility was higher when the consonant was in /a/ context (/a/ being the most represented phone in the corpus, see Fig 3A) than when in /i/ and /u/ context (*P* < 0.01), and no significant difference was observed between /i/ and /u/ contexts (*P* > 0.99): for instance, for *Pitch_27*, accuracy decreased from 80% for /a/ context, to 63% and 67% for /i/ and /u/ contexts respectively. Regarding consonants (Fig 6C), no clear differences were observed between the three synthesis *Pitch_27*, *Pitch_14* and *Pitch_10* except for /p/, /l/, /d/, /g/ and /ʒ/. Clear differences between these three conditions and *Pitch_7* were observed for consonants /p/, /f/, /b/, /v/, /ʒ/, /m/, /n/, /r/ and /l/. Clear differences were also observed between *FixedPitch_27* and *Pitch_27* for the unvoiced consonants /p/, /t/, /k/, /f/, /s/, and /ʃ/. Conversely, no significant differences between *FixedPitch_27* and *Pitch_27* were found for all the voiced consonants, which includes all the consonants chosen for the real-time closed loop synthesis that does not use the glottal activity (i.e. it is similar to *FixedPitch_27*). All conditions taken together, best results (at least one condition above 90%) were achieved for the fricative consonants /f/, /s/, /ʃ/, /z/, and /ʒ/, the nasal consonants /m/ and /n/, and /l/. Worst results (all conditions below 50%) were achieved for the plosive consonants /p/, /t/, /k/, /b/ and /d/. Note that there is no clear correlation with the number of occurrences of each phone in the training corpus, since for instance the corpus contained few instances of /ʃ/, and a large number of /t/ (Fig 3A).

**Fig 7:**
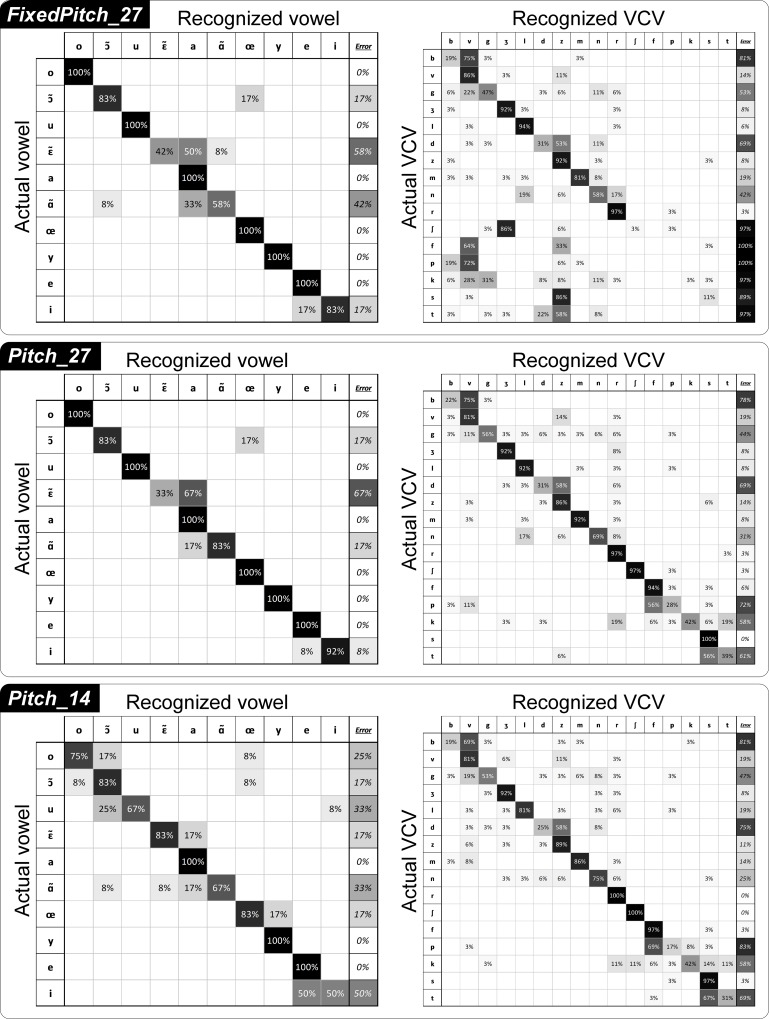
Confusion matrices of the subjective evaluation of the intelligibility of the speech synthesizer (offline reference synthesis). Confusion matrices for vowels (left) and consonants (right), for each of the three conditions FixedPitch_27, Pitch_27 and Pitch_14. In the matrices, rows correspond to ground truth while columns correspond to user answer. The last column indicates the amount of errors made on each phone. Cells are colored by their values, while text color is for readability only.

Analysis of the confusion matrices can enlighten the sources of synthesis errors (Fig 7). Each row *i* of a confusion matrix *M* corresponds to the ground truth phone *p*_*i*_, while column *j* corresponds to the phone *p*_*j*_ recognized by the listeners, so that a diagonal value *M*_*i*,*i*_ corresponds to the proportion of occurrences of the phone *p*_*i*_ that were correctly recognized, and a value *M*_*i*,*j*_ outside the diagonal corresponds to the proportion of occurrences of the phone *p*_*i*_ that were recognized as the phone *p*_*j*_. The order of the rows and columns of the confusion matrices were automatically sorted in order to emphasize the main confusions by forming high value blocks near the diagonal.

The confusion matrices of the perceptual listening test for the condition *Pitch_27* (Fig 7, middle row) reflect the global good quality of this synthesis (indicated by the fact that they are near-diagonal matrices). For vowels, six out of the ten vowels were always correctly recognized (/o/, /u/, /a/, /œ/, /y/ and /e/). Main errors come from confusions between /$\tilde{ɛ}$/ and /a/ (67% of / $\tilde{ɛ}$ / were recognized as /a/), and other errors come from confusions between /$\tilde{ɑ}$/ and /a/ (17% of /$\tilde{ɑ}$/ were recognized as /a/), and between /$\tilde{ɔ}$/ and /œ/ (17% of /$\tilde{ɔ}$/ were recognized as /œ/. For consonants, main confusions came from /b/ being recognized as /v/ (75%), /d/ being recognized as /z/ (58%), /p/ being recognized as /f/ (56%) and /d/ being recognized as /z/ (58%). Other more minor errors come from /g/ being recognized as /v/ (11%), and /k/ being recognized as /r/ (19%) and /t/ (19%).

By comparing confusion matrices of *Pitch_27* with those of *FixedPitch_27*, we can observe that not using the glottal activity resulted in increased confusions mainly for the vowel /$\tilde{ɑ}$/ (accuracy going from 83% for *Pitch_27* to 58% for *FixedPitch_27*) while no clear difference can be observed for the other vowels. Note that between the two conditions *Pitch_27* and *FixedPitch_27*, the articulatory-to-acoustic model remains the same, the only change being the excitation signal that is used for the final synthesis with the MLSA filter. Importantly, for the consonants, not using the glottal activity resulted in a drastic decrease in the recognition accuracy of all the unvoiced consonants /p/, /t/, /k/, /f/, /s/ and /ʃ/, while all the voiced consonants remained recognized with similar accuracy. Indeed, /p/ was mainly recognized as /v/ (72%), /t/ as /z/ (58%), /f/ as /v/ (64%), /s/ as /z/ (86%), and /ʃ/ as /ʒ/ (86%). Note that /v/ is the voiced counterpart of /f/, /z/ of /s/ and /ʒ/ of /ʃ/. Hence, the use of the template-based excitation naturally leads to a predictable shift of the unvoiced consonants to their more or less corresponding (in terms of place of articulation) voiced counterparts.

By comparing the confusion matrices of *Pitch_27* with those of *Pitch_14*, we can observe that there is no clear pattern of increased confusions. This confirms the results previously obtained from Fig 6, where no significant differences between *Pitch_27* and *Pitch_14* were found for both vowels and consonants.

**Fig 8:**
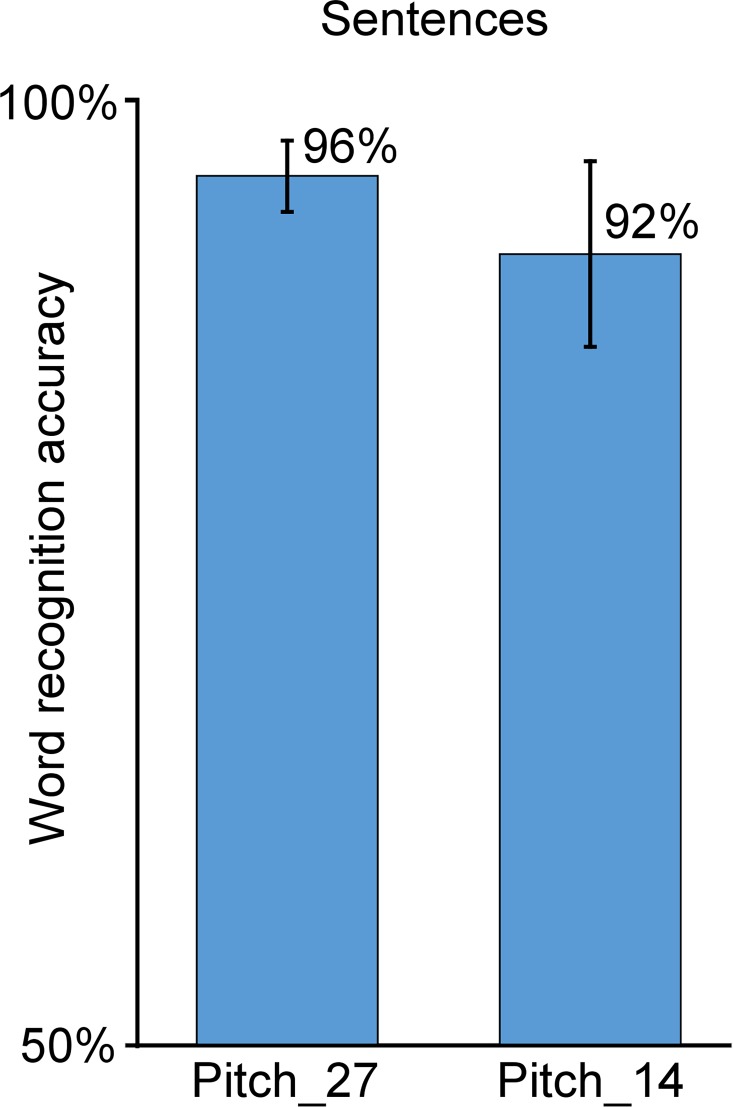
Subjective evaluation of the intelligibility of the speech synthesizer on sentences (offline reference synthesis). Word recognition accuracy for the sentences, for both conditions Pitch_27 and Pitch_14.

Finally, the results of the subjective evaluation on sentences are presented in Fig 8. While the recognition accuracy for *Pitch_27* and *Pitch_14* was below 90% for vowels and below 70% for consonants, the word recognition accuracy for the sentences is above 90% for both conditions (96% for *Pitch_27* and 92% for *Pitch_14*). Note that the difference in recognition accuracy for *Pitch_27* and for *Pitch_14* is here significant (*P* = 0.015).

### Control of the articulatory-based speech synthesizer (real-time closed-loop synthesis)

#### Accuracy of the articulatory-to-articulatory mapping

**Fig 9:**
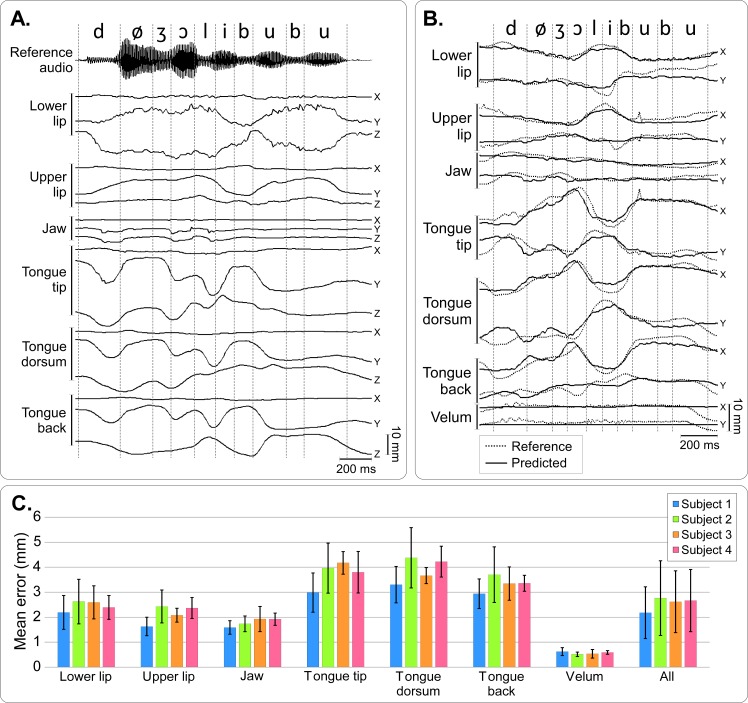
Articulatory-to-articulatory mapping. A–Articulatory data recorded on a new speaker (Speaker 2) and corresponding reference audio signal for the sentence “Deux jolis boubous” (“Two beautiful booboos”). For each sensor, the X (rostro-caudal), Y (ventro-dorsal) and Z (left-right) coordinates are plotted. Dashed lines show the phonetic segmentation of the reference audio, which the new speaker was ask to silently repeat in synchrony. B–Reference articulatory data (dashed line), and articulatory data of Speaker 2 after articulatory-to-articulatory linear mapping (predicted, plain line) for the same sentence as in A. Note that X,Y,Z data were mapped onto X,Y positions on the midsagittal plane. C–Mean Euclidean distance between reference and predicted sensor position in the reference midsagittal plane for each speaker and each sensor, averaged over the duration of all speech sounds of the calibration corpus. Error bars show the standard deviations, and “All” refer to mean distance error when pooling all the sensors together.

Fig 9A shows an example of articulatory data recorded from a new speaker (for instance Speaker 2), with the corresponding reference audio signal that the speaker was presented and asked to silently repeat synchronously (in this example, the sentence was *“Deux jolis boubous”*, meaning *“two beautiful booboos”*, which was not part of the training set). Fig 9B shows the transformation of these signals after their articulatory-to-articulatory mapping onto the reference speaker’s articulatory space. One can clearly see that articulatory movements of the new speaker were originally quite different than those of the reference speaker; and that they became similar once the articulatory-to-articulatory mapping was performed.

We further quantified the quality of the articulatory-to-articulatory mapping. Since articulatory data consist of geometrical coordinates, the mean Euclidean distance between predicted and true positions could be estimated for each sensor and for each speaker (Fig 9C). The average error across all sensors and speakers was 2.5 mm ± 1.5 mm. Errors were significantly higher for tongue sensors than for non-tongue sensors (*P* < 0.005 for 22 out of 24 pairwise comparisons corrected for multiple comparisons–see Methods), and lower for the velum sensor than for the non-velum sensors (*P* < 0.001 for 10 out of 12 pairwise comparisons corrected for multiple comparisons–see Methods). This is consistent with the fact that the tongue and velum are the articulators for which movement amplitudes were the highest and lowest, respectively (see Fig 3B). Mean distances for the reference speaker (Speaker 1) were systematically lower than for other speakers for all sensors except the velum. These differences were statistically significant for the tongue tip (*P* = 0.00229) and the tongue dorsum (*P* = 0.03051).

#### Intelligibility of the real-time closed-loop speech synthesis

**Fig 10:**
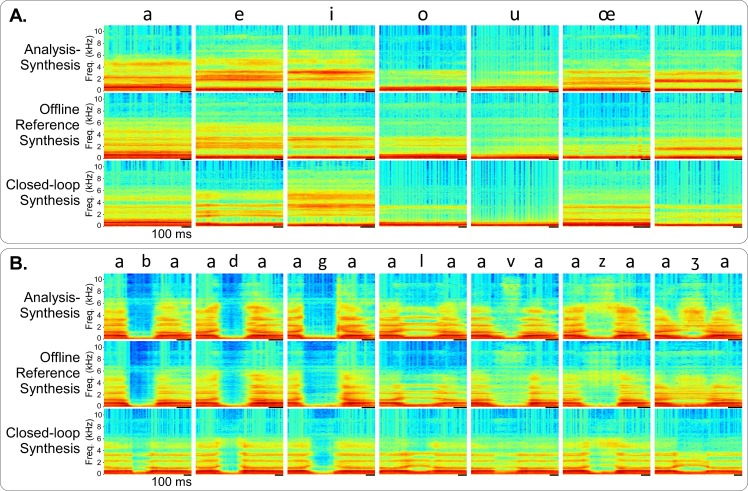
Real-time closed loop synthesis examples. Examples of audio spectrograms for anasynth, reference offline synthesis and real-time closed-loop (Speaker 2), A) for the vowels /a/, /e/, /i/, /o/, /u/, /œ/ and /y/, and B) for the consonants /b/, /d/, /g/, /l/, /v/, /z/ and /ʒ/ in /a/ context. The thick black line under the spectrograms corresponds to 100 ms.

During real-time control, the speakers were asked to reproduce a specific set of test sounds (see Materials and Methods). The remaining time of the experiment was kept for other tasks, including spontaneous conversations. S1 and S2 Video Files illustrate the closed-loop experiment, with Speaker 1 (reference speaker) and Speaker 2 (new speaker), respectively. Fig 10 shows examples of spectrograms of vowels and VCVs obtained during a session of real-time control (Speaker 2, first occurrence of each sound), compared with the corresponding spectrograms of anasynth and reference offline synthesis sounds. In general, we found that the spectrograms for the three conditions presented very similar characteristics, although some differences did exist in their fine structure, especially for consonants. For instance, the real-time examples of the plosive consonants /b/, /d/ and /g/ showed more energy smearing from vocalic to consonant segments as compared to the anasynth and offline synthesized versions. Also, the real-time example of /ʒ/ had characteristics closer to the anasynth version of /l/ than to the anasynth version of /ʒ/ (Fig 10B).

**Fig 11:**
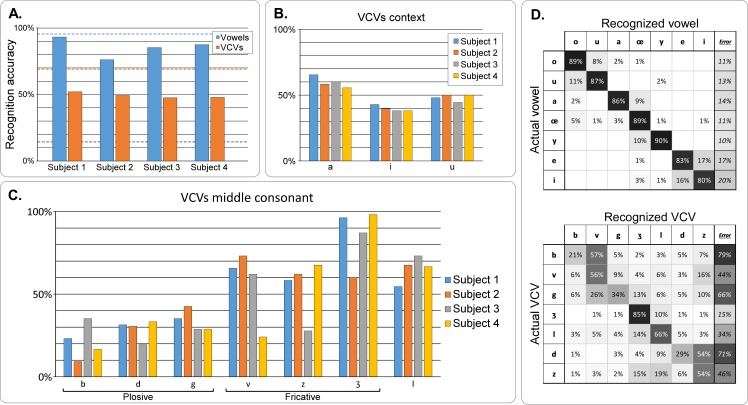
Results of the subjective listening test for real-time articulatory synthesis. A–Recognition accuracy for vowels and consonants, for each subject. The grey dashed line shows the chance level, while the blue and orange dashed lines show the corresponding recognition accuracy for the offline articulatory synthesis, for vowels and consonants respectively (on the same subsets of phones). B–Recognition accuracy for the VCVs regarding the vowel context, for each subject. C–Recognition accuracy for the VCVs, by consonant and for each subject. D–Confusion matrices for vowels (left) and consonants from VCVs in /a/ context (right). Rows correspond to ground truth while columns correspond to user answer. The last column indicates the amount of errors made on each phone. Cells are colored by their values, while text color is for readability only.

The test sounds produced in the closed-loop experiment were recorded and then their intelligibility was evaluated in the same way as for the offline synthesis intelligibility evaluation, i.e. a subjective intelligibility test performed by 12 listeners. Fig 11 summarizes the result of this listening test. The speech sounds produced by all 4 speakers obtained high vowel accuracy (93% for Speaker 1, 76% for Speaker 2, 85% for Speaker 3, and 88% for Speaker 4, leading to a mean accuracy score of 86%), and reasonable consonant accuracy (52% for Speaker 1, 49% for Speaker 2, 48% for Speaker 3, and 48% for Speaker 4, leading to a mean accuracy score of 49%). These scores were far above chance level (chance = 14%, *P* < 0.001) for both vowels and consonants. For all speakers, the 48–52% VCVs accuracy obtained during real-time control is to be compared to the 61% score obtained for the same VCVs in the offline reference synthesis. The difference is significant (*P* = 0.020 for reference speaker and *P* < 0.001 for other speakers, compare Fig 11A and Fig 6A) but the decrease is quite limited when considering that the speaker is no longer the reference speaker and that the synthesis is performed in an online closed-loop condition. The same observation applies to the vowel identification results: The 76–93% vowel accuracy for the closed-loop online synthesis is also found significantly lower than the 99% accuracy score obtained for the same vowels in the offline synthesis (*P* < 0.001 for reference and other speakers), but the decrease is relatively limited. The recognition accuracy for vowels was significantly higher for the reference speaker (*P* = 0.002) but no significant difference between the reference speaker and the other speakers was found for the VCVs (*P* = 0.262), even if the reference speaker obtained the highest average accuracy value for VCVs.

Regarding the vocalic context (Fig 11B), VCVs in /a/ context had better recognition accuracy than those in /i/ context (*P* < 0.001) and /u/ (*P* < 0.001) for all subjects, which is consistent with results from the offline reference synthesis. VCVs in /u/ context were found to have a better recognition accuracy than those in /i/ context (*P* = 0.009). Regarding the VCVs (Fig 11C), the recognition accuracy varied largely across consonants, ranging from an average of 21% for /b/ to 85% for /ʒ/. It was generally lower for the plosive consonants /b/, /d/ and /g/, which is consistent with results from the offline reference synthesis, while the accuracy on the remaining consonants was different for each subject. For instance, Subjects 1, 2 and 4 had good accuracy on /v/ while Subject 4 had a much lower accuracy. Similar result can be observed for /z/ and /ʒ/ for different subjects.

Confusion matrices for both vowels and consonants are shown in Fig 11D. These confusion matrices present features that are similar to the confusion matrices obtained for offline articulatory synthesis (Fig 7), and reflect well the results quality. All vowels show a recognition accuracy above 80%, and the highest accuracy was obtained for /y/, with 90%. The majority of the confusions are between /e/ and /i/ (17% of /e/ were recognized as /i/, and 16% of /i/ as /e/). Secondary confusions are between /o/ and /u/ (11% of /u/ were recognized as /o/, and 8% of /o/ as /u/), between /y/ and /œ/ (10% of /y/ were recognized as /œ/), and between /a/ and /œ/ (9% of /a/ were recognized as /œ/). The confusion matrix for consonant roughly corresponds to the confusion matrix obtained for offline articulatory synthesis, with emphasized confusions. Thus, the main confusions occurred again for plosive consonants /b/ (57% of /b/ were recognized as /v/) and /d/ (54% of /d/ were recognized as /z/), while quite few errors were made on /ʒ/ (85% of accuracy). Some errors were also made on /g/ but with less systematic confusion (26% with /v/, 13% with /ʒ/, and 10% with /z/). However, new confusions appeared that explain the significant drop in consonants accuracy with respect to offline articulatory synthesis: between /ʒ/ and /l/ (10% of /ʒ/ were recognized as /l/), and between /z/ and /l/ (19% of /z/ were recognized as /l/).

## Discussion

In this paper we first presented an articulatory-based speech synthesizer built from articulatory-acoustic data from one reference speaker using deep neural networks, and then showed that this synthesizer could be controlled in real-time closed-loop situation by several speakers using motion capture data (electromagnetic articulography) as input parameters. Experiments included the same reference speaker in a different session, as well as other speakers. All speakers were silently articulating and were given the synthesized acoustic feedback through headphones. A calibration method was used to take into account articulatory differences across speakers (and across sessions for the reference speaker), such as sensor positioning and ways of articulating the different sounds. Subjective listening tests were conducted to assess the quality of the synthesizer and in particular its performance during real-time closed-loop control by new speakers.

We first assessed the intelligibility of the synthesizer itself. Several versions of the synthesizer were built to assess the effect of the number of articulatory parameters (27, 14, 10 and 7), and the effect of using or not using glottal activity (by comparing synthesis using a constant artificial pitch, and the original pitch). The phone recognition accuracy for offline reference synthesis was far above chance level for all five tested parameterizations, and fully intelligible speech sentences could be produced (see Fig 8 and S1–S5 Audio Files). Most errors on vowels were made between vowels that had close articulatory positions (e.g. /i/ and /e/, see Fig 3B). Regarding consonants, most errors were made on the plosive consonants, and main confusions were observed within pairs of consonants corresponding to relatively similar articulatory movements in terms of place of articulation: for instance, /b/ is a labial consonant and /v/ is a labio-dental, and /d/ is a dental or an alveolar and /z/ is an alveolar. For /b/-/v/ confusion, this could be explained by a positioning of the EMA coils too far from the lip edges, resulting in a tracking of the lips by the EMA system that did not allow to capture sharp differences between /b/ and /v/ lip movements. A similar interpretation can be given for /d/-/z/ confusions, since in practice the coil had to be attached more than 5 mm back from the tongue tip (see Fig 2A). Moreover, results showed that the accuracy on the VCVs was correlated to the vocalic context, with consonant in /a/ context having a better recognition accuracy. This could be explained by the fact that the phone /a/ is more largely present in the training corpus than the phones /i/ and /u/ (see Fig 3A). However, this is not consistent with the fact that some phones that are less represented in the corpus, like /ʃ/, have high recognition accuracy, while other phone that are largely represented, like /d/, have low recognition accuracy. Another possible explanation is that /a/ is the most opened vowel and thus VCVs in /a/ context are performed by movements of higher amplitude, which could be more discriminant. By removing the glottal activity information (here by using a constant pitch), we found that the recognition accuracy significantly decreased for all unvoiced consonants, while remaining roughly the same for all voiced consonants and vowels (see Fig 6 and top and middle rows of Fig 7). The unvoiced consonants were thus confused with their voiced counterpart (e.g. /ʃ/ with /ʒ/), or with the voiced counterpart of the consonant they were already confused with (for instance, /p/ was originally confused with /s/ in the *Pitch_27* condition and was then confused with /ʒ/ when using a constant pitch, in the *FixedPitch_27* condition). This supports the choice we made to keep only 7 consonants for the real-time closed-loop synthesis since no glottal activity was available in silent speech condition. It should be noted that the quality of the synthesis could still be improved by estimating an optimal MLSA excitation source using inverse glottal filtering and that it could be envisioned that the parameters of such supplementary signal be predicted from brain signals in a BCI situation.

Regarding the number of articulatory parameters, results showed that using 14 articulatory parameters yields intelligibility scores that are close to the best scores achieved with 27 parameters. This supports the choice that was made to use a synthesizer with 14 articulatory parameters for the real-time closed-loop synthesis. Interestingly, using 10 parameters did not significantly impact the intelligibility of consonants, but started to affect that of vowels, although the accuracy remained at the high level of 67%. Decreasing further the number of parameters down to 7, significantly impacted the intelligibility of both vowels and consonants. Finally, although the accuracy on consonants was inferior to 70% for 27 and 14 articulatory parameters, this was enough to produce very intelligible sentences, with word recognition accuracy superior to 90% (see Fig 9). This can be explained by the fact that most confusions were made with similar consonants, thus ensuring a good intelligibility when constrained with closed vocabulary and syntactic rules. Thus, overall, the number of parameters required to achieve a sufficient intelligibility is of the order of the 10 degrees of freedoms that could be controlled successfully in recent state of the art BCI experiments (Wodlinger et al. 2015). It should be noted that the reduction in the number of parameters was done here in a drastic way either by dropping parameters or by PCA, while more efficient dimensionality reduction techniques could be envisioned such as autoencoders that we previously started to investigate [35].

Next, we assessed the intelligibility of the real-time closed-loop synthesis. In this case, the phone recognition accuracy was again far above chance level, both for vowels and consonants (Fig 11). Interestingly, this good intelligibility was obtained despite significant trajectory errors made on input control parameters obtained by the articulatory-to-articulatory mapping (about 2.5 mm on average, see Fig 9B). This confirms our previous results indicating that DNN-based articulatory synthesis is robust to fluctuations of the input parameters [35]. As expected, the closed-loop synthesis intelligibility was lower than for the reference offline synthesis. However, it was relatively limited. Confusions were similarly distributed in both cases, indicating that using the synthesizer in a closed-loop paradigm mainly emphasized the already existing confusions. The fact that most errors were consistent between offline and closed-loop synthesis suggests that real-time closed-loop articulatory synthesis could still benefit from improving the articulatory-to-acoustic mapping. This could be achieved by efficiently detecting specific constrictions from the articulatory data in order to improve the synthesis of plosive consonants, which are the major source of errors. The presence of additional minor confusions suggests that other aspects might also be improved, such as the articulatory-to-articulatory mapping with a better calibration approach. Indeed, to remain in a situation as close as possible to future BCI paradigms with aphasic participants, the articulatory-to-articulatory calibration step was performed under a silent speech condition. This was also consistent with the fact that the closed-loop condition was also performed in a silent speech condition so that the speaker received only the synthesized feedback, not superimposed on his/her own produced speech. Thus the articulatory-to-articulatory mapping converted articulatory trajectories recorded under a silent speech condition (for each speaker) into articulatory trajectories recorded under overt speech condition (of the reference speaker). Previous studies have shown that articulatory movements differ between silent and overt speech, and especially that silent speakers tend to hypo-articulate [57,58]. Such phenomenon may thus leads to smaller discrimination of articulatory trajectories during silent speech.

Improving the articulatory-to-articulatory and the articulatory-to-acoustic mappings might however not be the sole possibility to improve the intelligibility of closed-loop speech synthesis. Indeed, while results from the evaluation of the articulatory-to-articulatory mapping showed that for most sensors the mean prediction error was lower for Speaker 1 (the reference speaker), the results obtained during the real-time experiment showed that other speakers could achieve a control of the articulatory synthesizer similar to Speaker 1, in particular for consonants (see Fig 11A). For example, episodes of spontaneous conversation could be achieved not only with Speaker 1 but also with Speaker 2 (see S1 and S2 Video Files). This suggests that other factors come into play for the control of the synthesizer. One possibility is that subjects may adapt differently to the articulatory-to-articulatory mapping errors and find behavioral strategies to compensate for these errors. Here, each subject had about 20 minutes of free closed-loop control of the synthesizer between the two productions of test items, but we could not see any significant improvement over this short period of time. Finding behavioral strategies might thus need a more significant amount of training time. Finally, and according to the results from the offline reference synthesis, all unvoiced consonants were excluded since no glottal activity can be recorded in silent speech condition. In a BCI application for speech rehabilitation, such glottal activity could be predicted from the neural activity, thus allowing the synthesis of all the French phones.

To our knowledge these results are the first indication that an intelligible articulatory-based speech synthesizer can be controlled in real-time by different speakers to produce not only vowels, but also intelligible consonants and some sentences (some spontaneous conversations, while not reported here, could be achieved with 2 of the 4 subjects using only the synthesized audio i.e. the interlocutor could not see the subject articulating). These results thus go beyond previous preliminary achievements of speech synthesis from EMA data where discrete sentences could be successfully classified in a closed vocabulary context with training and testing performed in the same subjects [59]. Indeed, here the speech synthesis was performed in real time on a frame-by-frame basis to provide an online audio feedback delivered in real time with a very short time delay (less than 30 ms). Moreover, we showed here that a synthesizer built from a reference speaker data in an overt speech condition could be controlled to produce free speech in real time in a silent speech condition by other speakers with a different vocal tract anatomy and a different articulatory strategy using a simple linear calibration stage. This result is of particular interest for the emerging research field on ‘silent speech interfaces’, which are lightweight devices able to capture silent articulation using non-invasive sensors and convert it into audible speech [60–63]. Indeed, although the presented EMA-based interface is not strictly a silent-speech interface, the present results indicate that it is possible to synthesize intelligible speech in real time from articulatory data acquired in silent speech condition. Further studies could extend these results using less invasive techniques to obtain articulatory signals, such as EMG [61,62] and/or ultrasound signals [63,64].

Finally, this study is also a first step toward future speech BCI applications. Here we indeed showed that closed-loop speech synthesis was possible by subjects that had different speech production constrains (e.g., different anatomy of the vocal tract, different manner of articulation) than those of the reference speaker from whom the speech synthesizer was built. This means that differences in anatomical constrains could be compensated by the articulatory-to-articulatory mapping. In the context of a speech BCI paradigm, a similar situation will be encountered, where the synthesizer will be built from a subject different that the BCI participants. In this case, the question will be whether differences in neuronal constrains between individuals can also be compensated by a proper neural signal decoding strategy. Here, the DNN-based mapping approach was robust to trajectory errors of several millimeters that were present in the input signals of the synthesizer resulting from imperfections in the articulatory-to-articulatory mapping. This is encouraging given that decoding neural signal into input signals of the synthesizer will also be imperfect, and suggests that an articulatory-based speech synthesizer such as the one developed and tested here is a good candidate for being used in a speech BCI paradigm. The choice we made here to envision articulatory parameters as an intermediate representation for decoding speech from neural activity recorded from the speech motor cortex. This hypothesis will need to be tested in future BCI experiment and compared to a direct decoding of cortical activity into acoustic speech parameters.

## Supporting Information

S1 Appendix List of the sentences used for the subjective evaluation of the synthesizer. (DOCX)

S1 Audio File Example of original audio from the reference speaker. The corresponding sentence is *“Le fermier est parti pour la foire”* (*“The farmer went to the fair”*).
(WAV)

S2 Audio File Example of a sentence synthesized from the reference speaker data (reference offline synthesis) for the *FixedPitch_27* condition (fixed-pitch template-based excitation signal and 27 articulatory parameters). The corresponding sentence is *“Le fermier est parti pour la foire”* (*“The farmer went to the fair”*).
(WAV)

S3 Audio File Example of a sentence synthesized from the reference speaker data (reference offline synthesis) for the *Pitch_27* condition (excitation signal generated using the pitch extracted from the original audio and 27 articulatory parameters). The corresponding sentence is *“Le fermier est parti pour la foire”* (*“The farmer went to the fair”*).
(WAV)

S4 Audio File Example of a sentence synthesized from the reference speaker data (reference offline synthesis) for the *Pitch_14* condition (excitation signal generated using the pitch extracted from the original audio and 14 articulatory parameters). The corresponding sentence is *“Le fermier est parti pour la foire”* (*“The farmer went to the fair”*).
(WAV)

S5 Audio File Example of a sentence synthesized from the reference speaker data (reference offline synthesis) for the *Pitch_10* condition (excitation signal generated using the pitch extracted from the original audio and 10 articulatory parameters). The corresponding sentence is *“Le fermier est parti pour la foire”* (*“The farmer went to the fair”*).
(WAV)

S6 Audio File Example of a sentence synthesized from the reference speaker data (reference offline synthesis) for the *Pitch_7* condition (excitation signal generated using the pitch extracted from the original audio and 7 articulatory parameters). The corresponding sentence is *“Le fermier est parti pour la foire”* (*“The farmer went to the fair”*).
(WAV)

S1 Video File Example of spontaneous conversation during the real-time closed-loop control of the synthesizer by the reference speaker (Speaker 1). The corresponding sentence is *“Je ne t’entends pas”* (*“I cannot hear you”*).
(MP4)

S2 Video File Example of spontaneous conversation during the real-time closed-loop control of the synthesizer by a new speaker (Speaker 2). The corresponding sentence is *“Je vais être papa*. *C’est une bonne occasion de vous l’annoncer*. *Je suis très content*.*”* (*“I am going to be a father*. *It is a good opportunity to tell you this*. *I am very happy*.*”*).
(MP4)
