# Supplementary material for: Real-Time Control of an Articulatory-Based Speech Synthesizer for Brain Computer Interfaces
Source: PLoS Comput Biol. 2016 Nov 23;12(11):e1005119. doi: 10.1371/journal.pcbi.1005119 (PMC5120792; doi:10.1371/journal.pcbi.1005119)
Supplement: S1 Appendix — (DOCX) [file pcbi.1005119.s001.docx]

1. Francfort a reculé de un virgule quarante et un pourcent.
2. Au moins une sévère leçon.
3. Les préparatifs vont bon train.
4. Comme un regain en somme.
5. Allègre portrait d'un tueur psychopathe.
6. Les prix flambent dans l'hôtellerie.
7. Vous êtes un homme d'appareil.
8. Les travaux des bûcherons.
9. Le reste sera européen.
10. Le trafic d'être humain augmente.
11. Leur avenir semble aussi incertain.
12. Encore moins à son affiche.
13. Tel est le message implicite.
14. Et la clôt fort dignement.
15. Un homme toujours de dos.
16. Elle se trompe de débat.
17. Il y en a un.
18. Avec grandeur et densité.
19. Voilà où nous en sommes.
20. Elle en épaissit les outrances.
21. Les besoins y sont énormes.
22. Le quinze de France.
23. Ne pas rater le coach.
24. Le dollar chute, l'euro s'envole.
25. J'en suis totalement incapable.
26. Les autres réorganisations devraient suivre.
27. Les gammes familiales s'étoffent.
28. L'œuf du serpent aurait éclos.
29. Jugement le dix-neuf janvier.
30. Il lui faudra huit ans.
